# Clinical Significance of the Head-Up Tilt Test in Improving Prognosis in Patients with Possible Neurally Mediated Syncope

**DOI:** 10.3390/biology10090919

**Published:** 2021-09-15

**Authors:** Kengo Ayabe, Tomoyoshi Komiyama, Misaki Hasegawa, Tetsuri Sakai, Masahiro Morise, Susumu Sakama, Atsuhiko Yagishita, Mari Amino, Yuji Ikari, Koichiro Yoshioka

**Affiliations:** 1 Department of Cardiology, Tokai University School of Medicine, Isehara 259-1193, Kanagawa, Japan; 299947@cc.u-tokai.ac.jp (M.H.); st219820@tsc.u-tokai.ac.jp (T.S.); m-morise@tokai.ac.jp (M.M.); ss014572@tsc.u-tokai.ac.jp (S.S.); ayagishita@tsc.u-tokai.ac.jp (A.Y.); mariam@is.icc.u-tokai.ac.jp (M.A.); ikari@is.icc.u-tokai.ac.jp (Y.I.); ko1@is.icc.u-tokai.ac.jp (K.Y.); 2 Department of Clinical Pharmacology, Tokai University School of Medicine, Isehara 259-1193, Kanagawa, Japan

**Keywords:** atrial fibrillation, atrioventricular block, hypotension, sinoatrial node, tilt-table test, vasovagal syncope

## Abstract

**Simple Summary:**

Several diseases can cause syncope, which is commonly known as fainting; however, syncope triggered by a reflex mechanism, also termed neurally mediated syncope (NMS), is one of the most common forms. While NMS is considered a benign disease, it can cause critical clinical events, such as severe trauma due to syncope. The head-up tilt test (HUTT) is one of the modalities for the diagnosis of NMS. The clinical significance of HUTT in the prognosis of NMS, such as recurrence rate of syncope and mortality, are still to be elucidated. This research aimed to clarify the value of HUTT for the diagnosis of NMS, and to investigate the prognosis of patients with NMS by analyzing the data of 101 patients with syncope and their close long-term (four years at the longest) follow-up. Furthermore, as insertable cardiac monitors (ICMs) are considered effective in patients with syncope, this study also aimed to evaluate the usefulness of ICM in patients with negative HUTT results. Finally, our research contributes to the improvement of the clinical management for patients with syncope.

**Abstract:**

Syncope is commonly encountered in daily clinical practice. Depending on its etiology (benign or life-threatening conditions or environmental triggers), syncope can be neurally mediated (reflex), cardiac, or orthostatic. Furthermore, neurologic disease can cause symptoms that mimic syncope. However, there is limited research on neurally mediated syncope (NMS), which is considered a benign disorder, and close follow-ups are rarely performed. NMS can cause serious clinical events, including severe trauma and car accidents. The head-up tilt test (HUTT) is the gold standard for diagnosing NMS; however, its clinical significance remains unknown, and its relevance to NMS prognosis requires further research. This retrospective study aimed to assess the clinical significance of the HUTT for NMS. We reviewed the charts of 101 patients who underwent HUTT at Tokai University Hospital in Japan between January 2016 and March 2019. During the HUTT, 72 patients (69.2%) experienced syncope. Patients were followed up for 886.1 ± 457.7 days (interquartile range: 518–1293 days). The syncope recurrence rate was 16.9%; however, no significant difference was observed between the two groups (HUTT positive vs. negative) (13.8% vs. 18.1%, *p* = 0.772). Four of 29 (13.9%) and two of 72 (2.8%) patients in the negative and positive HUTT groups, respectively, experienced cardiac events (*p* = 0.019). Negative HUTT results may assist in anticipating unexpected clinical events within a few years. A negative HUTT result may allow us to reconsider the NMS diagnosis based on clinical information. Close outpatient follow-up of patients with negative HUTT results is warranted.

## 1. Introduction

Syncope is a common chief complaint encountered in daily clinical practice. It is defined as the transient loss of consciousness due to global cerebral hypoperfusion [1], which is characterized by a rapid onset and spontaneous recovery. Depending on the underlying etiology (benign or life-threatening conditions or environmental triggers), syncope can be neurally mediated (reflex), cardiac, or orthostatic. Furthermore, some neurologic diseases can cause symptoms that mimic syncope [2]. Although the severity and clinical significance of syncope might vary according to the patient’s background, cardiogenic syncope was found to be associated with poor prognosis [3,4]. The main causes of cardiogenic syncope are ischemic heart diseases, valvular diseases, and life-threatening arrhythmias, such as ventricular tachycardia, ventricular fibrillation, and conduction disorders (complete heart block and sick sinus syndrome). On the contrary, neurally mediated syncope (NMS) is non-life-threatening and represents a benign disorder with a better prognosis [5]. NMS comprises a relatively wide variety of types, such as vasovagal, situational, carotid sinus, and others [3]. To clarify the cause of syncope, circulatory dynamics are usually investigated in patients with NMS using high-resolution Holter electrocardiography and correlation analysis of changes in adenylate cyclase activity, blood pressure, and pulse during the head-up tilt test (HUTT) [6,7]. Additionally, the mechanism of the molecular interaction and the polymorphisms of the alpha-2 adrenoreceptor (α2B-AR) gene as the potential psychiatric cause of incentive stress are analyzed [8]. 

Despite its benign nature, NMS prevents patients from performing their regular daily activities due to the unanticipated onset of the disease and a paucity of treatment options [9]. Therefore, the categorization of NMS as a benign disorder is questionable, and further research is warranted. 

While obtaining the details of clinical history is very important, the HUTT is one of the standard diagnostic procedures for NMS [10]. According to the guidelines on syncope published by the Japanese Society of Cardiology (2012), European Society of Cardiology, and American College of Cardiology and American Heart Association, NMS is classified into four types (vasopressor, cardioinhibitory, mixed, and orthostatic hypotension types) based on the results of this test [2,11]. The clinical importance of the HUTT has been studied widely. However, the clinical sensitivity and specificity of the HUTT have not yet been adequately established [12,13]. Herein, we focus on the clinical significance of HUTT in patients with syncope and whose probable diagnosis was NMS. Limited studies concerning the long-term follow-up of patients with syncope have been reported [14]. The prognosis of NMS remains to be elucidated. Therefore, we followed up our patients for a maximum of 3 years. The specificity of the HUTT has been reported to be as high as 90% [3,15]. Hence, we also focused on the clinical importance of negative HUTT results because close follow-up of these patients is occasionally not performed due to the belief that NMS is a benign disorder. Negative HUTT results suggest that the etiology of syncope remains to be determined by more detailed examination. Insertable cardiac monitors (ICMs) are widely used in patients with syncope of unknown etiologies [16]. In addition, ICMs are effective in detecting atrial fibrillation (AF), one of the many causes of syncope due to sinus node pause [17]. Although we did not perform ICM implantation in our patients, we also aimed to evaluate the usefulness of ICM in patients with syncope whose HUTT results were negative.

## 2. Materials and Methods

### 2.1. Ethics Statement

The Institutional Review Board of Tokai University School of Medicine approved our study (approval number: 14R-053). 

### 2.2. Patient Information and Data Collection

We performed a retrospective chart review of 101 patients who underwent the HUTT at Tokai University Hospital in Japan between January 2016 and March 2019. 

All patients presented with syncope once or more times at our hospital, and were initially evaluated by a cardiologist. Laboratory analyses, electrocardiography (ECG), echocardiography, and chest radiography were also performed to rule out cardiac diseases, such as coronary artery disease and cardiomyopathy. Echocardiography was performed to rule out systolic and diastolic dysfunctions and structural abnormalities using standard methods. Additionally, electroencephalography and computed tomography of the head were performed in some patients to rule out epilepsy. NMS was diagnosed based on systemic inquiry, thoughtful clinical history taking, and clinical tests. HUTT was performed in patients whose most likely diagnosis was NMS. Patients with any possibility of cardiac or neurological causes were excluded from the study. Patients’ information, including age, past medical history, history related to syncope, and social history, were collected from their medical charts.

### 2.3. Head-Up Tilt Test (HUTT)

**Figure 1:**
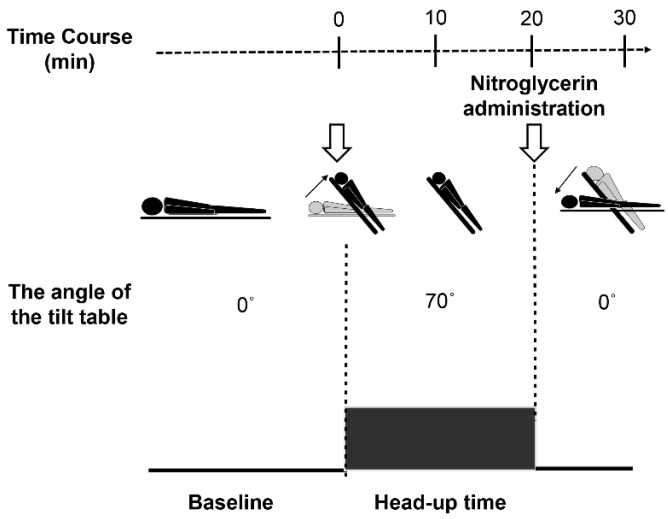
HUTT protocol.

The HUTT was performed as previously described [6]. All study patients were admitted to our hospital a day before the test. We performed the test in the morning after sufficient night rest. The tilt table was set to a standing position at 70 degrees (70°). The patient initially lay on the table and wore belts to prevent accidental injury if syncope occurred. We then placed a continuous blood pressure monitoring and ECG electrode patch on the patient, using the tilt test monitoring device developed by Nihon Koden (Nihon Kohden, Tokyo, Japan). Blood pressure, heart rate, and ECGs were monitored continuously. The room light and air conditioner were turned off during the test. After confirming that the patient was in a stable condition with his/her security belts on, we moved up the study patient to 70°. The patient was instructed to stand for 20 min before administering nitroglycerin sublingually. Afterwards, the patient was instructed to stand for another 10 min. When the patient complained of presyncopal symptoms or developed syncope, we defined the HUTT result as positive. If syncope occurred, the tilt bed was leveled immediately, and vital signs were maintained. The protocol of the HUTT is shown in Figure 1.

This figure illustrates the HUTT protocol. HUTT was performed till the end without any syncope. If syncope occurred, the tilt table was moved back to 0° immediately. If syncope did not occur for 20 min, nitroglycerin was sublingually administered. The total time for HUTT was 30 min.

### 2.4. Evaluation of the HUTT Results

Blood pressure, heart rate, and ECG were continuously monitored during the test. We classified the HUTT results into four types according to the latest guidelines for syncope published by the Japanese Society of Cardiology: vasopressor, cardioinhibitory, mixed, or orthostatic hypotension. The cardioinhibitory type was defined as a heart rate of <40 beats/min that lasted for 10 s, or cardiac arrest lasting >3 s before syncope. The vasopressor type was characterized by a reduction in blood pressure without bradycardia. The mixed type was comprised of both of the above-mentioned characteristics. Classical orthostatic hypotension was defined as a reduction in blood pressure that was sufficient to cause syncope within 3 min of a change in position from supine to standing. A positive test result was defined as the occurrence of presyncope or syncope during the test, regardless of the necessity of sublingual nitroglycerin administration. We calculated each percentage of the four NMS types listed above and analyzed the patients’ data for each NMS group. An unexpected cardiac event was defined as a cardiac issue that was not noticeable at the point of the HUTT, and required an invasive procedure or hospital admission for further treatment. Sudden deaths were also included in this category.

### 2.5. Follow-Up in the Patients after the HUTT

Patients were instructed to perform the tilt training at home by standing against a wall twice a day for a planned duration of up to 30 min, regardless of the HUTT results. The effectiveness of this tilt training was recommended by a previous study [18]. Patients were followed up for a maximum of 3 years at an outpatient clinic regarding syncope recurrence. Additionally, data of any unexpected events, such as death or unanticipated syncopal problems, were collected. If patients did not present to the outpatient clinic, we contacted them asking the same questions that were asked at the clinic.

### 2.6. Statistical Analyses

All statistical analyses were performed using the statistical software EZR (Saitama Medical Center, Jichi Medical University, Saitama, Japan), which is a graphical user interface for R (The R Foundation for Statistical Computing, Vienna, Austria). More precisely, it is a modified version of R commander designed to add statistical functions frequently used in biostatistics [19]. Log-rank analysis and a Mann–Whitney U test were used to analyze the data.

## 3. Results

### 3.1. Patients’ Characteristics

Between March 2016 and June 2019, we performed 101 HUTTs in the patients whose probable diagnosis was NMS based on their clinical history and diagnostic tests, such as blood tests, ECG, 24-h Holter electrocardiography, and echocardiography, performed by cardiologists at our institute (Table 1).

There was no significant difference in baseline patient characteristics between the positive HUTT group and the negative HUTT group.

The median age was 49.6 ± 21.0 years and there were 67 male patients (66.3%). Patients with cardiac diseases, such as ischemic heart disease and conduction disease that required immediate treatment were excluded. Regarding blood test results of all patients, hemoglobin levels and electrolyte levels, such as sodium, potassium, and calcium, were within normal limits. Of all included patients, 28.9% had hypertension. The study included patients who were on renin-angiotensin system inhibitors (20.2%), α-blockers (3.9%), β-blockers (8.65%), diuretics (5.8%), and calcium channel blockers (16.4%). Twelve patients (11.5%) had diabetes mellitus, which was well controlled with medications. Two patients (2.0%) had a family history of NMS.

Before the HUTT, we confirmed that the patients’ cardiac condition was stable enough to tolerate the test. The mean left ventricle ejection fraction measured using echocardiography was 67.7 ± 9.0%. There was no significant difference in the electrocardiographic findings between the positive and negative HUTT groups. One patient had a history of epilepsy. She was evaluated by a neurologist, and no association between syncope and epilepsy was confirmed. All patients presented with syncope without any other provocation.

### 3.2. HUTT Result

**Figure 2:**
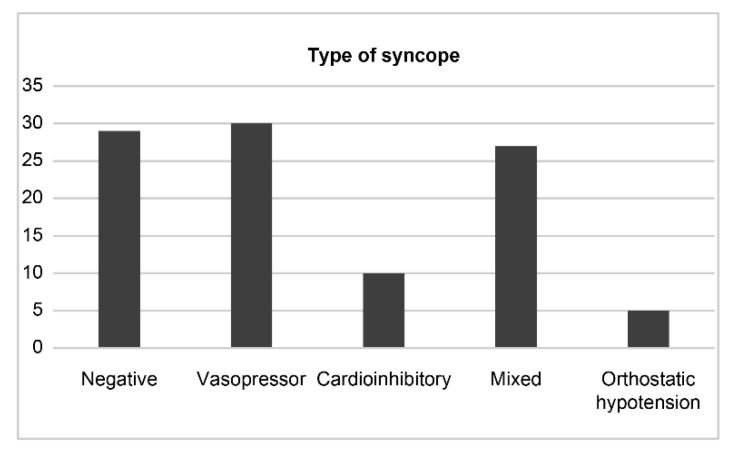
HUTT results are classified according to the NMS type. Negative included the patients who did not presented with syncope during HUTT.

During the HUTT, 72 patients (69.2%) experienced syncope. No adverse events (AEs), except for syncope, were documented during the test. Blood pressure, heart rate, or both dropped when the patient experienced syncope. However, the patients were managed appropriately by the bedside physician. The types of syncope are shown in Figure 2. The syncope was classified as vasopressor, cardioinhibitory, mixed type, and orthostatic hypotension in 30 (27.9%), 10 (9.6%), 27 (25.9%), and five (4.8%) patients, respectively (Figure 2). There was no significant difference between the two groups in terms of the use of medications listed in Table 1, presence of a history of cardiac diseases, diabetes mellitus, hypertension, AF, and stroke. There was no significant difference in age (51.8 ± 19.7 vs. 48.8 ± 21.6 years, *p* = 0.59) and left ventricle ejection fraction (64.9 ± 10.7 vs. 68.8 ± 8.1%, *p* = 0.13) between the two groups.

### 3.3. Details of 12 Lead ECG Analysis

We performed analysis of 12 lead ECG of each patient who underwent HUTT. The only significant differences were shorter QRS intervals and QT intervals in patients with a positive HUTT compared to that of those with a negative HUTT (Table 2).

### 3.4. Recurrence of Syncope after the HUTT and ICM Placement

**Figure 3:**
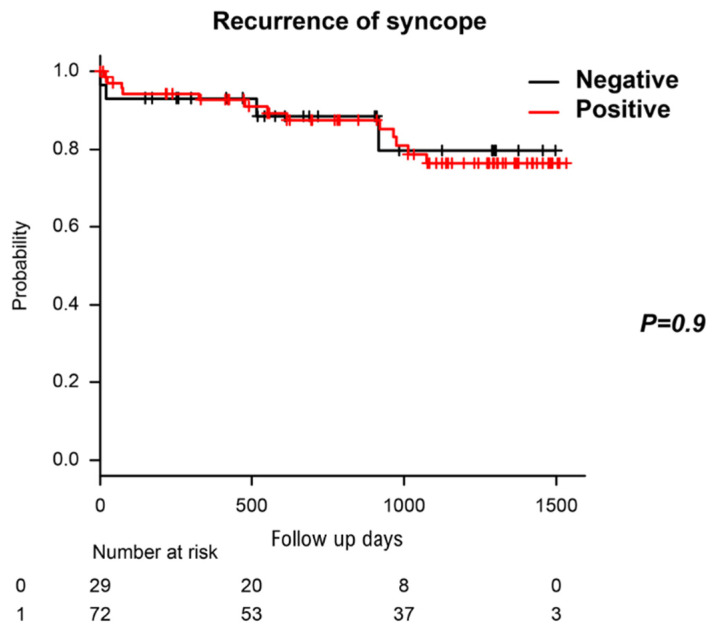
Kaplan Meier curves depict the recurrence rate of syncope according to the HUTT results. The black line stands for the patients whose HUTT was negative. The red line stands for the patients whose HUTT was positive.

The patients were followed up for 886.1 ± 457.7 days (interquartile range: 518–1293 days). The recurrence rate of syncope was 16.9%, and there was no significant difference between the two groups in recurrence rate (13.8% vs. 18.1%, *p* = 0.772, Figure 3). We performed log-rank analysis and found no significant difference between the two groups (*p* = 0.772).

Two patients (6.9%) in the negative HUTT group underwent ICM placement. However, no significant arrhythmia was detected to explain the cause of syncope. None of the patients in the positive HUTT group underwent ICM placement, and none of the patients received any prescription for the treatment of NMS.

### 3.5. Unexpected Cardiac Events after the HUTT

**Figure 4:**
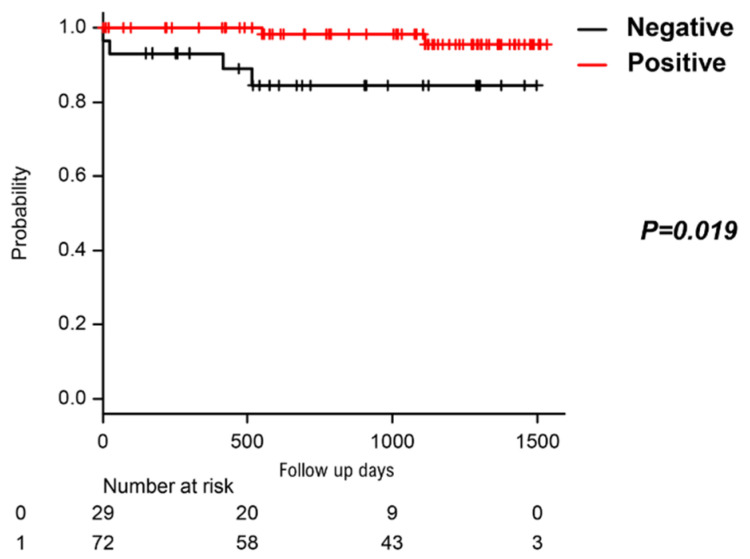
KAPLAN Meier curves depict the occurrence rate of unanticipated cardiac events classified according to the HUTT results. The black line stands for the patients whose HUTT was negative. The red line stands for the patients whose HUTT was positive.

Regular follow-up of patients revealed six unexpected cardiac events (5.9%). Four out of 29 (13.9%) patients in the negative HUTT group and two out of 72 (2.8%) in the positive HUTT group experienced cardiac events. Of the four patients in the negative HUTT group, one patient required a pacemaker implantation due to an advanced atrioventricular conduction block. One patient required an implantable cardioverter defibrillator for syncope associated with ventricular tachycardia due to possible sarcoidosis. One patient underwent catheter ablation for AF with a rapid ventricular response (RVR). Sudden death occurred in the last patient 417 days after the HUTT. Among the two patients in the positive HUTT group, one patient required pacemaker implantation due to sick sinus syndrome, and the other required hospital admission because of AF with RVR. Details of all events are listed in Table 3. We performed log-rank analysis and found significant differences in the occurrence rate of cardiac events between the two groups (*p* = 0.019, Figure 4).

## 4. Discussion

This retrospective clinical study assessed the clinical significance of the HUTT in patients with NMS. The results demonstrated that vasopressors and mixed types of NMS were more common than the cardioinhibitory type. While the pre-test probability was high, the rate of positive test results was not as high as anticipated. Although we ruled out possible diseases that could cause syncope before the test, the patients’ condition on the test date might have affected the test results. There are several studies on the HUTT, and our results are comparable to previous research data [20]. Sublingual nitroglycerin administration during the HUTT has also made the results obtained more accurate [21]

NMS is sometimes underestimated in daily clinical settings because its prognosis is believed to be benign. Outpatient follow-up is sometimes discontinued at the patient’s or physician’s discretion [22]. A limited variety of treatment options, such as medications and tilt training, have also contributed to inadequate outpatient follow-up [23]. Therefore, there was a paucity of studies of syncope recurrence after the HUTT. We followed up our patients for a relatively long period and found that the recurrence rate was 16.9%. It has been reported that syncope recurrence in the elderly was as high as 32.5% [22]. Since our patients had a median age of 49.6 ± 21.0 years, the recurrence rate was lower than the average rate reported in previous studies [24]. Of note, there was no significant difference between the negative and positive HUTT result groups. We did not compare our patients with syncope to patients who did not undergo the HUTT. However, the test itself and education for syncope may help patients avoid unexpected syncope recurrence in their daily life. We educated all included patients to perform the tilt training after they were discharged home from the hospital. Although there are conflicting results regarding the efficacy of the tilt training, a high compliance rate with this training in our patients may have contributed to the low recurrence rate of syncope [18,22]. More research is needed to elucidate how a high compliance rate with tilt training can improve the rate of syncope recurrence. None of our patients received pharmacological treatment because there is a lack of evidence for the efficacy of pharmacological treatment in patients with NMS [25].

Although there have been several reports regarding the usefulness of the HUTT in the last decade, we suggest that two important findings regarding the clinical significance of the test should be noted [20,26]. First, if the test is positive, we could confirm that the cause of syncope is NMS. It is very important to rule out other etiologies of syncope before the test. It has been reported that 15.5% of all patients with syncope admitted to the emergency department (ED) experienced AEs within a year [27]. It should be noted that the etiology itself was unknown when patients present with syncope at the ED for the first time and that patients who are brought to the ED tend to be in a more critical condition than those who visit the outpatient clinic. Therefore, it is reasonable that the occurrence rate of AEs was higher in patients who presented to the emergency room than in our study patients. If a patients HUTT results are negative, such a patient should not be diagnosed with NMS. However, we found that patients with negative HUTT results could encounter unexpected cardiac events more frequently than those with positive HUTT results. Therefore, close outpatient monitoring, including 24-h Holter electrocardiography and blood tests, should be recommended. In addition, ICM is also recommended for unexplained syncope.

All current guidelines published in the United States and Europe are in general agreement that the decision to use an ICM for syncope should consider patients’ characteristics, frequency of syncope, and pre-test probability of arrhythmic cause [28]. Several studies reported that ICM placement led to a 36.6–51.8% of diagnostic yield during the 17–39 months of follow-up period [29,30]. Our findings suggest that a negative HUTT requires further investigation to determine the etiology of syncope. All our patients presented with unexpected cardiac events. Arrhythmia related to AF is a common cause of syncope among the elderly [31]. Therefore, patients with negative HUTT results and older age should undergo ICM implantation for possible intracardiac conduction disorder and AF.

### Limitations

This was a single-center study with a small number of patients. Due to the retrospective nature of the study, selection bias could not be excluded. It has been reported that female patients might develop NMS more frequently than their male counterparts [32]. Thus, our study does not reflect the real-world scenario. Further research with a larger number of patients is required to validate our findings. Closer attention should have been paid to the characteristics of patients with syncope. Our patients were selected because they had a high likelihood of NMS to take the HUTT. Therefore, selection bias cannot be ignored. In other words, although the initial diagnosis appears to be NMS, we should endeavor to determine the etiology of syncope when the HUTT is negative. We found that negative HUTT result is associated with more cardiac events; however, further studies are needed to support this finding.

While ICM is recommended as per the current guidelines for unknown etiology syncope, only two patients in our study underwent ICM placement. This may affect the analysis of the recurrence rate of NMS.

## 5. Conclusions

Negative HUTT results may assist in anticipating unexpected clinical events within a few years in patients with syncope. A negative HUTT result may allow us to reconsider the diagnosis of NMS based on clinical information. However, close outpatient follow-up for patients with negative HUTT results is warranted.

## Figures and Tables

**Table 1:** Baseline clinical characteristics of the study patients.

	Negative (n = 29)	Positive (n = 72)	*p* Value
Age	51.8 ± 19.7 years old	48.8 ± 21.6 years old	0.51
Male	72.4% (21)	63.9% (46)	0.49
Hypertension	41.4% (12)	25% (18)	0.15
RAS inhibitor	27.6% (8)	18.1% (13)	0.49
α blocker	3.4% (1)	4.2% (3)	1
β blocker	13.8% (4)	6.9% (5)	0.27
Diuretics	13.8% (4)	7.8% (2)	0.06
Calcium channel blocker	20.7% (6)	15.3% (11)	0.56
CVA	3.4% (1)	0% (0)	0.29
Diabetes mellitus	13.8% (4)	11.1% (8)	0.74
Atrial fibrillation	6.8% (2)	5.5% (4)	1
Ischemic heart disease	13.8% (4)	6.9% (5)	0.27
Non-ischemic heart disease	6.8% (2)	1.4% (1)	0.2
Ejection fraction of Left ventricle	64.9 ± 10.7%	68.8 ± 8.1%	0.05
History of Smoking	51.7% (15)	43.1% (31)	0.31
Epilepsy	0% (0)	1.4% (1)	1

Abbreviations: CVA, Cerebrovascular attack; RAS, renin-angiotensin system.

**Table 2:** Analysis of baseline electrocardiogram.

	HUTT		*p* Value
	Negative (n = 29)	Positive (n = 71)	
HR	68 ± 14	62 ± 14	0.06
PR	163 ± 30.6	162 ± 23.8	0.88
Axis	65.2 ± 79.2	46.3 ± 39.0	0.1
QRS	113 ± 61.9	94.4 ± 19.3	0.03
QTc	430 ± 33.9	413 ± 26.9	0.01
RBBB	3 (10.3%)	10 (14.1%)	0.75
LBBB	2 (6.9%)	0 (0%)	0.08
Brugada	1 (3.4%)	2 (2.8%)	-
DP	0 (0%)	1 (1.4%)	-
ERP	3 (10.3%)	13 (18.3%)	0.547

Abbreviations: HR, heart rate (beats per minute); PR, PR interval (ms); Axis, electronic axis (degree); QRS, QRS interval (ms); RBBB, right bundle branch block; LBBB, left bundle branch block; DP, delayed potentials; ERP, early repolarization.

**Table 3:** Details of cardiac events in each patient.

Positive HUTT					
Patient	Sex	Age	The Type of NMS	Days after HUT Test	The Details of Cardiac Event
1	Female	70	Vasopressor	549	Although no AF was detected prior to HUTT, the patient presented with syncope due to AF with rapid ventricular response. She underwent catheter ablation because her AF was refractory to antiarrhythmic medications.
2	Female	73	Vasopressor	1111	Although no AF was detected prior to HUTT, the patient presented with syncope due to AF with rapid ventricular response. She was told to undergo catheter ablation, however, she refused the procedure.
**Negative HUT test**					
3	Female	75	N/A	23	The patient presented with syncope and found to have 5 sec of long pause with faintness at the emergency room. The patient required pacemaker implantation due to sick sinus syndrome.
4	Male	82	N/A	516	The patient presented with bradycardia and syncope and was brought to the emergency room. He was found out to have advanced atrioventricular conduction block and required pacemaker implantation.
5	Male	55	N/A	417	The patient had history of old myocardial infarction (ejection fraction of 44%) of left anterior descending artery. He was found to be dead at home due to unknown etiology. After the investigation, we decided that the most likely cause of death was cardiogenic disease.
6	Female	61	N/A	1	The patient presented with syncope and the monitor electrocardiogram demonstrated unstable ventricular tachycardia with heart rate was 180 beats per minute. The syncope occurred on the day when we performed HUTT. She was diagnosed as cardiac sarcoidosis and required ICD placement.

Abbreviations: HUTT, head-up tilt test; NMS, neurally mediated syncope; AF, Atrial Fibrillation, ICD, Implantable Cardioverter Defibrillator.

## Data Availability

Not applicable.
